# Diagnostic accuracy of pocket‐sized ultrasound for aspiration pneumonia in elderly patients without heart failure: A prospective observational study

**DOI:** 10.1111/ggi.14293

**Published:** 2021-10-14

**Authors:** Harumitsu Yamanaka, Hiroki Maita, Tadashi Kobayashi, Takashi Akimoto, Hiroshi Osawa, Hiroyuki Kato

**Affiliations:** ^1^ General Medicine Hirosaki University Graduate School of Medicine Aomori Japan; ^2^ Fujishiro‐Kensei Hospital Aomori Japan; ^3^ Development of Community Healthcare Hirosaki University Graduate School of Medicine Aomori Japan; ^4^ Department of General Medicine Hirosaki University School of Medicine & Hospital Aomori Japan

**Keywords:** aspiration pneumonia, diagnostic accuracy, elderly patients, lung diseases, ultrasound

## Abstract

**Aim:**

To investigate the diagnostic accuracy of pocket‐sized ultrasound (PsUS) for aspiration pneumonia in elderly patients without heart failure.

**Methods:**

This prospective observational study included patients with aspiration pneumonia. PsUS was performed in six areas (bilateral chest, four dorsal areas) by an independent examiner, blinded to the computed tomography (CT) results as a reference standard. Patients with heart failure were excluded.

**Results:**

PsUS findings of 34 patients (median age, 87.5 years) and 204 areas were analyzed. Three or more B‐lines (comet tail artifacts) were strongly suggestive (positive likelihood ratio [LR+] 17.302) of consolidation on CT (CT‐consolidation, subpleural hypoechoic area with tissue‐like echostructure) or pleural change on CT. Consolidation on US (US‐consolidation) was suggestive of CT‐consolidation or pleural changes on CT (LR+ 6.453). Pleural effusion on US was strongly suggestive (LR+ 10.989) of CT‐consolidation or pleural change on CT. Absence of either three or more B‐lines, US‐consolidation, or US pleural effusion could not rule out CT‐consolidation or pleural change on CT (negative likelihood ratio [LR−] 0.482‐0.683). However, absence of all three findings could rule out abnormal CT findings (LR− 0.230). Chest radiograph findings proved difficult to confirm or exclude CT‐consolidation or pleural changes on CT (LR+ 1.584, LR− 0.489); when combined with PsUS findings, LR− improved to 0.124.

**Conclusions:**

Three or more B‐lines or US‐consolidation on PsUS in elderly patients with aspiration pneumonia without heart failure suggested CT‐consolidation or pleural changes on CT. When both PsUS and chest radiograph findings were negative, CT‐consolidation and pleural change could be excluded. **Geriatr Gerontol Int 2021; 21: 1118–1124**.

## Introduction

In Japan, approximately 70% of patients with pneumonia are aged ≥75 years, and more than 70% of pneumonia cases in the elderly involve aspiration pneumonia.^1^ The diagnosis and treatment of aspiration pneumonia are challenging in countries with aging populations. Generally, pneumonia is diagnosed by new‐onset radiographic findings on simple chest radiographs;^2^ however, some areas cannot be evaluated sufficiently due to the overlap of the lung with other organs. Therefore, chest computed tomography (CT), which compensates for this disadvantage, is an excellent reference standard for pneumonia diagnosis.^3^, ^4^ However, CT is not available in all medical facilities because of its high cost and radiation exposure risk.

Lung ultrasound (US) is a new technique for diagnosing lung and pleural diseases; its diagnostic usefulness for pneumonia has been widely reported in emergency patients,^5^, ^6^ the elderly,^7^ adults^8^ and children.^9^ Recent technological innovations have given compact, high‐performance, and low‐cost portable US devices that facilitate early diagnosis and subsequent monitoring of aspiration pneumonia^10^ at home.^11^ Although the diagnostic accuracy of lung US for pneumonia in the elderly is reportedly superior to that of simple chest radiography,^7^ no study has evaluated the diagnostic accuracy of pocket‐sized US (PsUS) for aspiration pneumonia in the elderly.

Lung US is also used in diagnosing heart failure and has a diagnostic accuracy comparable with that of simple chest radiography.^12^ The number of B‐lines can be used to semiquantitatively assess the pulmonary edema degree in diagnosing heart failure;^12^ therefore, we considered it appropriate to exclude the influence of overlapping heart failure findings for aspiration pneumonia evaluation. In this study, we investigated the diagnostic accuracy (sensitivity [Sn], specificity [Sp] and likelihood ratio [LR]) of PsUS for aspiration pneumonia in elderly patients without heart failure, using chest CT as a reference standard.

## Methods

This prospective observational study was conducted at a community hospital (Kensei Hospital) in Hirosaki City, Japan, and was approved by the ethics committees of Hirosaki University (approval number 2017‐1111) and Kensei Hospital (approval number 2017‐04). All data were fully anonymized at data collection, and written informed consent was obtained from all participants.

### Patients

This study included patients admitted to Kensei Hospital between March 2018 and June 2019 with a diagnosis of aspiration pneumonia according to the Japanese Respiratory Society guidelines.^13^ The inclusion criteria were as follows: (i) pulmonary CT upon admission, and (ii) PsUS performed before the results of radiological imaging (pulmonary CT or simple chest radiograph) were obtained. The exclusion criteria were as follows: (i) lack of informed consent; (ii) at least one heart failure finding on physical or blood examination and a heart failure result on CT; (iii) antibiotic use before diagnosis; and (iv) lung US and chest CT performed >24 h apart, with marked changes between the two images.

### Heart failure diagnosis

Heart failure was defined as a condition with one or all the following findings: (i) pulmonary congestion or pleural effusion on CT; (ii) clinical findings such as significant leg edema; and (iii) laboratory findings such as elevated B‐type natriuretic peptide (≥500 pg/mL). Three physicians independently and comprehensively evaluated the patients for heart failure.

### Lung ultrasound

Lung US was performed using miruco (Convex Array Probe, 3.5 MHz; body, miruco; NIPPON SIGMAX Co., Ltd., Tokyo, Japan) by a single trained physician blinded to the results of simple chest radiography and CT at six frequently reported sites (Figure S1) for aspiration pneumonia^4^ (chest bilaterally in the supine position and four sites on the right and left lateral part of the back in the lateral position). The six subdivided US areas of the patients were a unique classification defined for this study, considering the predominant site of aspiration pneumonia and the efficiency of physician's daily practice.

**Figure 1:**
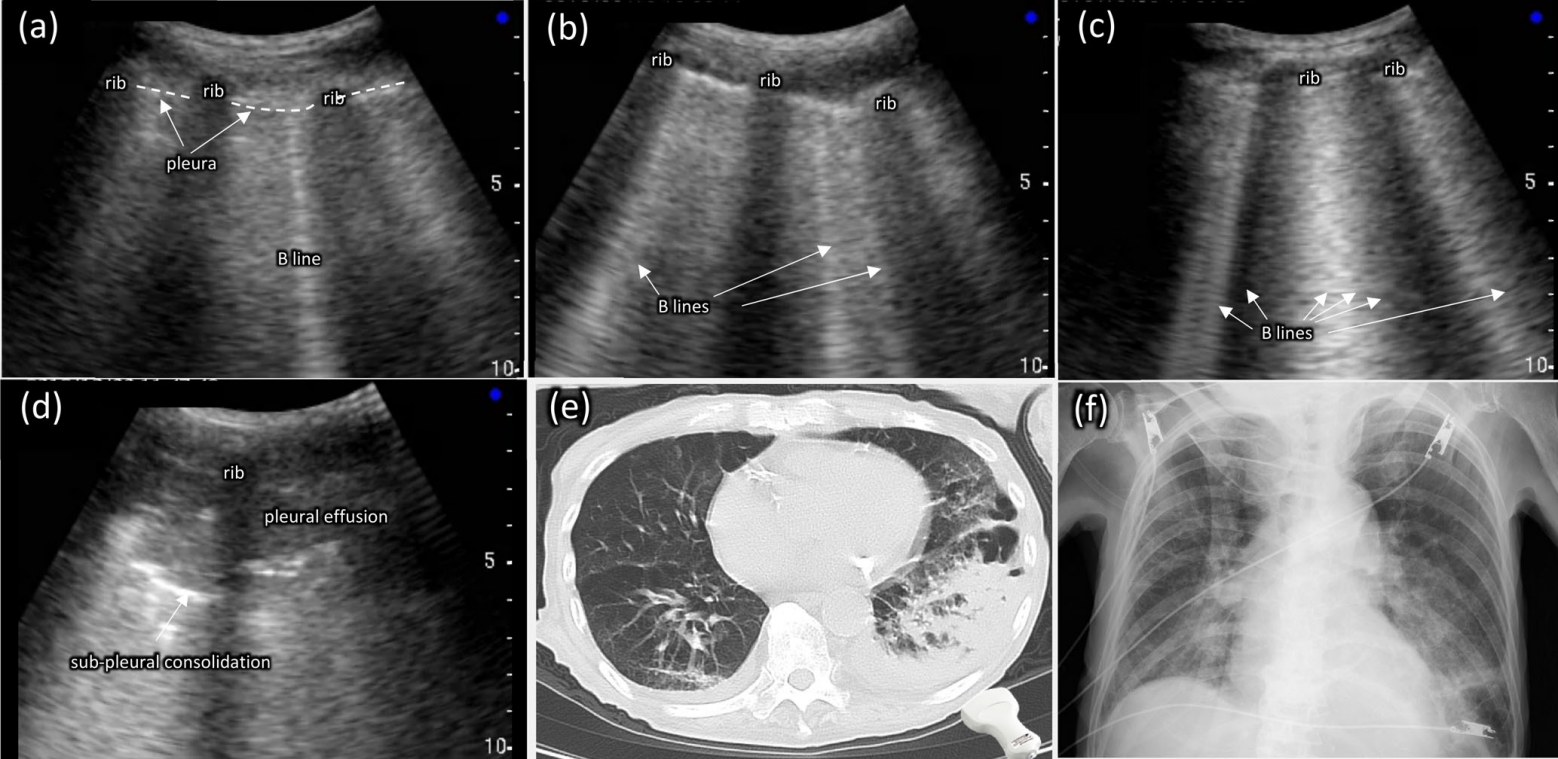
Representative images of US, simple chest radiography and CT. (a) One B‐line as US findings; (b) ≥3 B‐lines as US findings; (c) ≥5 B‐lines (including “white lung”) as US findings. (d) Consolidation and pleural effusion as US findings. (e) Typical CT findings of left‐sided aspiration pneumonia. US probe on the image shows the US image (d). (f) Typical chest radiographic findings of left‐sided aspiration pneumonia (loss of the silhouette of the left diaphragm arch behind the heart). (d–f) The images of (d), (e), and (f) are from the same patient. CT, computed tomography; US, ultrasound.

We compared the abnormal findings on lung US (B‐line, US‐consolidation, pleural effusion) (Fig. 1a–d) with those on CT (CT‐consolidation, pleural change) (Fig. 1e) and calculated Sn, Sp and LR. B‐line is a comet tail artifact and US‐consolidation is a subpleural hypoechoic area with echostructure resembling that of the liver, which could be observed as one of the sonographic features of pneumonia.^14^ Lung US images, recorded in miruco as digital images, were independently evaluated by three physicians. Abnormal findings on PsUS were confirmed based on consensus between the three physicians.

### Computed tomography imaging

Chest CT was used as a reference standard for aspiration pneumonia findings (CT‐consolidation and pleural changes). We defined CT‐consolidation as homogeneous opacities of the parenchyma obscuring the underlying vessels and little or no loss of volume, and pleural change as pleural thickening and pleural retraction. Clinical CT was performed for the diagnosis of aspiration pneumonia and heart failure by an independent radiologist. CT images in the area corresponding to the lung US findings were examined in scout image and axial view by three physicians independently of other examiners, who were blinded to the clinical findings and other imaging results. The decision on the existence of abnormal findings in each lung area was based on the consensus opinion of two or all three physicians.

### Chest radiography

Simple chest radiographs for the diagnosis of clinical aspiration pneumonia at the time of the visit to the emergency room or outpatient department were independently evaluated by physicians (Fig. 1f). We compared the simple chest radiographic findings with CT findings at the six aforementioned areas and calculated Sn, Sp and LR. The findings on each simple chest radiograph and CT were independently evaluated by three physicians blinded to the clinical findings and other imaging results. Simple chest radiographs were divided into the six corresponding parts of the US, printed and evaluated to prevent the influence of abnormal findings in adjacent sections (Fig. S2). Abnormal findings were confirmed by the consensus opinions of the three physicians.

### Data collection

The following patient information was collected using the records in the PsUS device and the electronic medical record; age, sex, axillary temperature, maximum axillary temperature, blood pressure, pulse rate, oxygen saturation, inspiratory oxygen concentration, quick SOFA score, Clinical Frailty Scale score, classification of aspiration pneumonia diagnosis (definite or suspected), history of aspiration pneumonia (first occurrence or recurrence), respiratory tract symptoms, heart failure, anterior leg edema, blood test findings (white blood cell count, C‐reactive protein, maximum C‐reactive protein, blood urea nitrogen, creatinine and brain natriuretic peptide), lung US findings, chest CT findings, simple chest radiograph findings, and time from lung US to CT scan (Table 1).

**Table 1:** Baseline characteristics of patients (*n* = 34)

Baseline patient characteristics on admission	Values
Age (years), median (IQR 25%, 75%)	88 (82, 93)
Sex (male), *n* (%)	19 (55.9)
Axillary temperature (°C), mean ± SD	37.8 ± 0.9
Maximum axillary temperature (°C; during hospitalization), mean ± SD	38.6 ± 0.8
Systolic blood pressure (mmHg), median (IQR)	124.5 (111.3, 157.5)
Diastolic blood pressure (mmHg), median (IQR)	77.50 (64.0, 90.5)
Pulse rate (beats/min), mean ± SD	94.7 ± 20.7
Respiratory rate (breaths/min), mean ± SD	23.5 ± 6.0
Oxygen saturation (%), mean ± SD	92.1 ± 5.0
Patients requiring oxygen administration *n* (%)	14 (41.2)
Quick SOFA (point), *n* (%)	
0	2 (5.9)
1	10 (29.4)
2	19 (55.9)
3	3 (8.8)
4	0 (0)
Clinical frailty scale (point), *n* (%)	
0	0 (0)
1	0 (0)
2	0 (0)
3	0 (0)
4	1 (2.9)
5	2 (5.9)
6	3 (8.8)
7	18 (52.9)
8	9 (26.5)
9	1 (2.9)
Classification of the clinical diagnosis of aspiration pneumonia according to the guidelines of the Japanese Respiratory Society, *n* (%)	
Definitive	27 (79.4)
Suspected	7 (20.6)
History of aspiration pneumonia, *n* (%)	
First episode	20 (58.8)
Recurrent	14 (41.2)
Respiratory symptoms, *n* (%)	
Yes	33 (97.1)
No	1 (2.9)
Pre‐tibial edema, *n* (%)	
Positive	1 (2.9)
Negative	33 (97.1)
Congestive heart failure findings on computed tomography, *n* (%)	
Positive	0 (0)
Negative	100 (100)
Immunosuppressant use, *n* (%)	1 (2.9)
White blood cell count (/μL), mean ± SD	10 596 ± 4406
C‐reactive protein (mg/dL), median (IQR)	6.7 (1.8, 9.8)
Maximum C‐reactive protein (mg/dL) (during hospitalization), median (IQR)	10.0 (5.4, 16.7)
Blood urea nitrogen (mg/dL), median (IQR)	18.3 (14.1, 25.9)
Creatinine (mg/dL), median (IQR)	0.68 (0.6, 0.8)
Brain natriuretic peptide (pg/mL), median (IQR), *n* = 17	129.8 (75.4, 189.7)
Time between ultrasound and computed tomography (h), mean ± SD	21.3 ± 17.1

IQR, interquartile range; SD, standard deviation.

### Statistical analysis

All statistical analyses were performed using EZR (version 1.41; Saitama Medical Center, Jichi Medical University, Saitama, Japan).^15^ The 2 × 2 contingency tables were analyzed to calculate the Sn, Sp and LR.

## Results

In total, 50 patients with aspiration pneumonia met the inclusion criteria between March 2018 and June 2019; 16 patients were excluded (nine patients did not consent, four had cardiac failure findings on CT, and three could have marked changes between CT and US images because of time lag). Consequently, 34 patients (median age, 87.5 years; male, 55.6%) were analyzed. The patients in the study were elderly with relatively severe respiratory symptoms who were at high risk of sepsis (Table 1). Two of these patients did not undergo simple chest radiography (Fig. S3).

### Diagnostic accuracy of B‐line detected on ultrasound

For CT‐consolidation, the positive LR (LR+) for ≥3 B‐lines was 3.496, which was not very high, whereas the LR+ for ≥5 B‐lines was 6.00, which was suggestive of pneumonia. The negative LR (LR−) of ≥3 B‐lines was 0.54, which was not useful for ruling out pneumonia (Table 2a). For a pleural change, the LR+ for ≥3 B‐lines was 10.14, strongly suggestive of pleural changes, while the LR− was 0.429, which was not useful for ruling out pleural changes (Table 2b). For CT‐consolidation or pleural change, the LR+ for ≥3 B‐lines was 17.302, which was highly suggestive of CT‐consolidation or pleural change; the LR− was 0.482, which was not useful for ruling out CT‐consolidation or pleural changes (Table 2c).

**Table 2:** Diagnostic accuracy of B‐line for indicating CT‐consolidation, pleural change, or both on chest CT

Cut‐off value of B‐line	Sn (95% CI)	Sp (95% CI)	LR+ (95% CI)	LR− (95% CI)
(a) Diagnostic accuracy of B‐line for indicating CT‐consolidation				
1	0.728 (0.637–0.807)	0.489 (0.382–0.597)	1.424 (1.131–1.795)	0.556 (0.385–0.803)
3	0.544 (0.448–0.637)	0.844 (0.753–0.912)	3.496 (2.100–5.822)	0.54 (0.434–0.673)
5	0.333 (0.248–0.428)	0.944 (0.875–0.982)	6 (2.463–14.618)	0.706 (0.614–0.811)
(b) Diagnostic accuracy of B‐line for indicating pleural change on chest CT				
1	0.731 (0.642–0.808)	0.506 (0.395–0.616)	1.48 (1.163–1.883)	0.532 (0.370–0.764)
3	0.597 (0.503–0.686)	0.941 (0.868–0.981)	10.143 (4.279–24.043)	0.429 (0.342–0.537)
5	0.336 (0.252–0.428)	0.965 (0.900–0.993)	9.524 (3.047–29.772)	0.688 (0.602–0.787)
(c) Diagnostic accuracy of B‐line for indicating CT‐consolidation or pleural change on chest CT				
1	0.719 (0.637–0.792)	0.554 (0.425–0.677)	1.613 (1.206–2.155)	0.507 (0.359–0.715)
3	0.532 (0.446–0.617)	0.969 (0.893–0.996)	17.302 (4.382–68.314)	0.482 (0.402–0.579)
5	0.302 (0.227–0.386)	0.985 (0.917–1.000)	19.64 (2.763–139.596)	0.709 (0.633–0.794)

CI, confidence interval; CT, computed tomography; LR+, positive likelihood ratio; LR−, negative likelihood ratio; Sn, sensitivity; Sp, specificity.

### Diagnostic accuracy of ultrasound‐consolidation

For CT‐consolidation, the LR+ of US‐consolidation was 4.079, which was weakly suggestive of CT‐consolidation. The LR− was 0.526, which was not useful for ruling out CT‐consolidation. For the pleural change, the LR+ of US‐consolidation was 4.091, which was weakly suggestive of pleural changes. The LR− was 0.541, which was not useful in ruling out pleural changes. For CT‐consolidation or pleural change, the LR+ of US‐consolidation was 6.453, which was suggestive of CT‐consolidation or pleural change. The LR− was 0.546, which was not useful in ruling out CT‐consolidation or pleural change. (Table 3).

**Table 3:** Diagnostic accuracy of US‐consolidation for indicating CT‐consolidation and pleural change on chest CT

CT finding	Sn (95% CI)	Sp (95% CI)	LR+ (95% CI)	LR− (95% CI)
CT‐consolidation	0.544 (0.448–0.637)	0.867 (0.779–0.929)	4.079 (2.347–7.090)	0.526 (0.424–0.653)
Pleural change	0.529 (0.436–0.622)	0.871 (0.780–0.934)	4.091 (2.298–7.283)	0.541 (0.439–0.665)
CT‐consolidation or pleural change	0.496 (0.411–0.582)	0.923 (0.830–0.975)	6.453 (2.735–15.229)	0.546 (0.456–0.653)

CI, confidence interval; CT, computed tomography; LR+, positive likelihood ratio; LR−, negative likelihood ratio; Sn, sensitivity; Sp, specificity.

### Diagnostic accuracy of pleural effusion detected on ultrasound

For CT‐consolidation, the LR+ of the pleural effusion on US was 4.737, which was a weakly suggestive finding for CT‐consolidation. The LR− was 0.685, which was not useful for ruling out CT‐consolidation. For the pleural change, the LR+ of the pleural effusion on US was 10.952, which was highly suggestive of pleural change. The LR− was 0.636, which was not useful in ruling out pleural changes. For CT‐consolidation or pleural change, the LR+ of pleural effusion on US was 10.989, which was strongly suggestive of CT‐consolidation or pleural change. The LR− was 0.683, which was not useful in ruling out CT‐consolidation or pleural change (Table 4).

**Table 4:** Diagnostic accuracy of ultrasonographic pleural effusion for CT‐consolidation and pleural change on chest CT

CT finding	Sn (95% CI)	Sp (95% CI)	LR+ (95% CI)	LR− (95% CI)
CT‐consolidation	0.368 (0.280–0.464)	0.922 (0.846–0.968)	4.737 (2.235–10.037)	0.685 (0.588–0.798)
Pleural change	0.387 (0.299–0.480)	0.965 (0.900–0.993)	10.952 (3.523–34.049)	0.636 (0.548–0.738)
CT‐consolidation or pleural change	0.338 (0.260–0.423)	0.969 (0.893–0.996)	10.989 (2.753–43.860)	0.683 (0.602–0.775)

CI, confidence interval; CT, computed tomography; LR+, positive likelihood ratio; LR−, negative likelihood ratio; Sn, sensitivity; Sp, specificity.

### Diagnostic accuracy of combined lung ultrasound findings (B‐lines, ultrasound‐consolidation and pleural effusion)

The LR− of all three findings (B‐lines, US‐consolidation and pleural effusion) for CT‐consolidation or pleural change was 0.230, which weakly ruled out the presence of pneumonia or pleural changes. Other analyses of lung US combinations are presented in the Supporting Information (Table S1a–c).

### Diagnostic accuracy of simple chest radiographs in combination with pulmonary ultrasound findings

For pneumonia or pleural changes, the LR+ of abnormal radiographic findings on simple chest radiographs was 1.584. The LR− was 0.489, indicating that these findings were not useful for either definitive or exclusionary diagnosis. The results of other simple chest radiograph analyses are provided in the Supporting Information (Table S2). The LR− of the combined abnormal chest radiographic findings and all three abnormal US findings (B‐lines, US‐consolidation and pleural effusion) improved to 0.124. Results of all possible combinations of radiographic and US findings are provided in the Supporting Information (Table S3a–c).

## Discussion

Our study evaluated the diagnostic accuracy of PsUS for aspiration pneumonia in the elderly without heart failure. An exclusion criterion was a time interval of >24 h between lung US and chest CT, and presentation of marked changes in the images over time (cases where the findings of lung US and chest CT findings were expected to be different due to the increased time interval between the acquisition of images). We postulated that fluid movement in pulmonary congestion has a greater impact on imaging findings over time, than inflammation in pneumonia. Therefore, we focused on patients with aspiration pneumonia without congestive heart failure, as the findings of the latter can confound the evaluation of the former, and tried to reduce the effect of the time interval between US and CT. According to reports, abnormal findings on simple chest radiographs of community‐acquired pneumonia did not completely disappear in many cases, even after 1 week to 10 days.^16^, ^17^ Recently, high diagnostic accuracy was reported for lung US performed within 48 h before and after CT for coronavirus disease 2019 (COVID‐19) pneumonia (Sn 92.1%, Sp 90%).^18^ Therefore, we considered pulmonary US and chest CT comparable and included them in our analysis.

B‐line can detect pleural changes more specifically (Table 2b,c), and we suggest that checking for B‐lines (three or more) first in a high‐risk population for aspiration pneumonia would accelerate diagnosis. Unlike binary data (e.g., presence or absence of US‐consolidation), B‐lines can acquire different Sn and Sp by changing the cut‐off value of the ordinal scale. We considered the optimal cut‐off value of B‐line 3, which is the maximum Youden index, as positive (Tables 2 and 4). However, the presence of ≥5 B‐lines can be used as a specific finding for definitive diagnosis, and the absence of any B‐lines as a sensitive finding for exclusion diagnosis.

Lung consolidation is caused by the replacement of gas‐filled lung tissue with anechoic or tissue‐like material due to infiltration of fluid or inflammatory cells, seen in pneumonia, pulmonary edema, lung contusion and neoplastic diseases. In this study, when US‐consolidation was detected, the LR+ was 6.453 for CT‐consolidation or pleural change on CT (Table 3). Theoretically, US‐consolidation does not directly imply pleural change on CT; it rather indicates a pleural change in the proximity of CT‐consolidation, and these findings were significantly related (Fisher's exact test, *P* < 0.01). In evaluating aspiration pneumonia, the variables of CT‐consolidation and pleural change could not be evaluated independently when present in the same area. Therefore, we used CT‐consolidation or pleural changes as the main outcome of our analysis.

The combined evaluation of multiple diagnostic tests is reportedly an effective tool in the diagnosis of emergency cases with a complaint of pleural pain.^6^ In our study, we focused on three findings, i.e., B‐line, US‐consolidation and pleural effusion on lung US. We hypothesized that if these multiple US findings could be simultaneously evaluated, we would specifically detect lung abnormalities (CT‐consolidation or pleural change) in patients with aspiration pneumonia. When all these findings were present, the LR+ was 15.00, highly suggestive of lung disease even without a simple chest radiograph. Screening tests should be highly sensitive to exclude target diseases. To meet this requirement, we evaluated a combination of multiple US findings. The LR− of all these three findings for CT‐consolidation or pleural change was 0.230, and it improved to 0.124 when simple chest radiograph findings were included. Although aspiration pneumonia cannot be sufficiently excluded by PsUS or simple chest radiography alone (Table S2), combining the findings of both examinations may sufficiently lower the likelihood of the disease. Furthermore, lung US does not cause radiation exposure, does not require transfer to the radiology department, and can be performed repeatedly at the bedside. Therefore, US may be a better choice for monitoring changes over time.

In Japan, the number of patients with aspiration pneumonia is expected to increase as the proportion of the aging population increases. Lung US is expected to be used in countries with scarce medical resources, those with aging populations, and those that expect efficient use of medical resources. Appropriate use of PsUS, which is portable, easy‐to‐use and less invasive, in medical institutions and at home and nursing care facilities, may reduce unnecessary visits to medical institutions. The usefulness of lung self‐US for monitoring COVID‐19 has been reported and is expected to reduce the unnecessary infection spread due to patient movement.^19^ Therefore, lung US is expected to achieve a more accurate aspiration pneumonia diagnosis and decision making, particularly in primary care medical institutions and facilities not equipped with CT, leading to improved treatment, thereby contributing to effective medical resource utilization in the community.

This study had some limitations. First, there was a time lag between lung US and chest CT as a reference standard (median 17 h). As this was an observational study, there were certain practical limitations; therefore, scheduling of image acquisition and future evaluation through interventional studies is desirable. Second, we could not evaluate US accuracy based on the patient's body mass index. The quality of US images is affected by subcutaneous fat, which influences the diagnosis. At the study hospital, the stretcher scales were not routinely used; therefore, it was difficult to analyze the data by considering the patient's weight. Third, this study was conducted with a small number of patients at a single institution in Japan, and the results may differ depending on the disease severity, the setting (e.g., clinic, nursing home), and diagnostic criteria; therefore, generalizations should be made with caution. In addition, further studies are needed to evaluate patients with aspiration pneumonia complicated by heart failure. Fourth, the Sn of lung US is reportedly 80%–90%. The low Sn in our study may be because of the limited evaluation time in clinical practice and the inability to obtain sufficient suitable positions for observation, as many patients were bedridden. In addition, as pleural effusion could change with body position, comparison of CT (supine position) and US (lateral position) findings might have affected the diagnostic accuracy. Re‐evaluation by multiple examiners in various clinical settings is needed. Evaluation of diagnostic accuracy at sites other than the US areas (six subdivisions of patients) analyzed in this study is also necessary in the future. Fifth, our study was evaluated using a single portable US device and needs to be validated with other devices as well.

In conclusion, for elderly patients with aspiration pneumonia without congestive heart failure, three or more B‐lines or US‐consolidation detected on PsUS in elderly patients with aspiration pneumonia without heart failure suggested CT‐consolidation or pleural changes on CT. When both PsUS and chest radiograph findings are negative, pneumonia and pleural disease can be ruled out. PsUS is an inexpensive, easy‐to‐use, non‐invasive test that can contribute to both definitive and exclusionary diagnosis; it can be used in countries with limited medical resources and in countries and regions where aspiration pneumonia is increasing due to aging and where rational and efficient medical resource use is expected.

## Disclosure statement

The authors declare no conflict of interest.

## Supporting information


**Figure S1.** Chest and back subdivisions of patients undergoing lung ultrasound in this study. LL, lower lateral (lower part of the lateral thorax between the anterior axillary line and the posterior axillary line); LP, lower posterior (lower part of the back below the inferior angle of the scapula and dorsal to the posterior axillary line); MP, middle posterior (middle part of the back medial to the scapula)

**Figure S2.** Chest subdivisions of patients undergoing simple chest radiograph in this study. A, Line running across the middle of the scapula. B, Line running through the lower edge of the scapula. C, Line running through the costophrenic angle. D, Line running along the most lateral rib. E, Midline of the thoracic vertebrae
F, Line running through the lateral third of the lung. G, Line running through half the height of the lung.

**Figure S3.** Flow diagram of the study population

**Table S1.** (a) Diagnostic accuracy of a combination of ultrasound findings (B‐line and US‐consolidation and pleural effusion) for suggesting CT‐consolidation. (b) Diagnostic accuracy of a combination of ultrasound findings (B‐line and US‐consolidation and pleural effusion) for suggesting pleural change on chest CT. (c) Diagnostic accuracy of a combination of ultrasound findings (B‐line and US‐consolidation and pleural effusion) for suggesting CT‐consolidation or pleural change on chest CT.

**Table S2.** Diagnostic accuracy of chest radiography for CT findings (CT‐consolidation and pleural change).

**Table S3.** (a) Diagnostic accuracy of a combination of simple chest radiography and ultrasound findings (B‐line and US‐consolidation and pleural effusion) for CT‐consolidation (b) Diagnostic accuracy of a combination of simple chest radiography and ultrasound findings (B‐line and US‐consolidation and pleural effusion) for pleural change on chest CT. (c) Diagnostic accuracy of a combination of simple chest radiography and ultrasound findings (B‐line and US‐consolidation and pleural effusion) for CT‐consolidation or pleural change on chest CT.

## Data Availability

The data that support the findings of this study are available from the corresponding author upon reasonable request.
