# Supplementary figures and images for: Diagnostic accuracy of pocket‐sized ultrasound for aspiration pneumonia in elderly patients without heart failure: A prospective observational study
Source: Geriatr Gerontol Int. 2021 Oct 14;21(12):1118–24. doi: 10.1111/ggi.14293 (PMC9293111; doi:10.1111/ggi.14293)

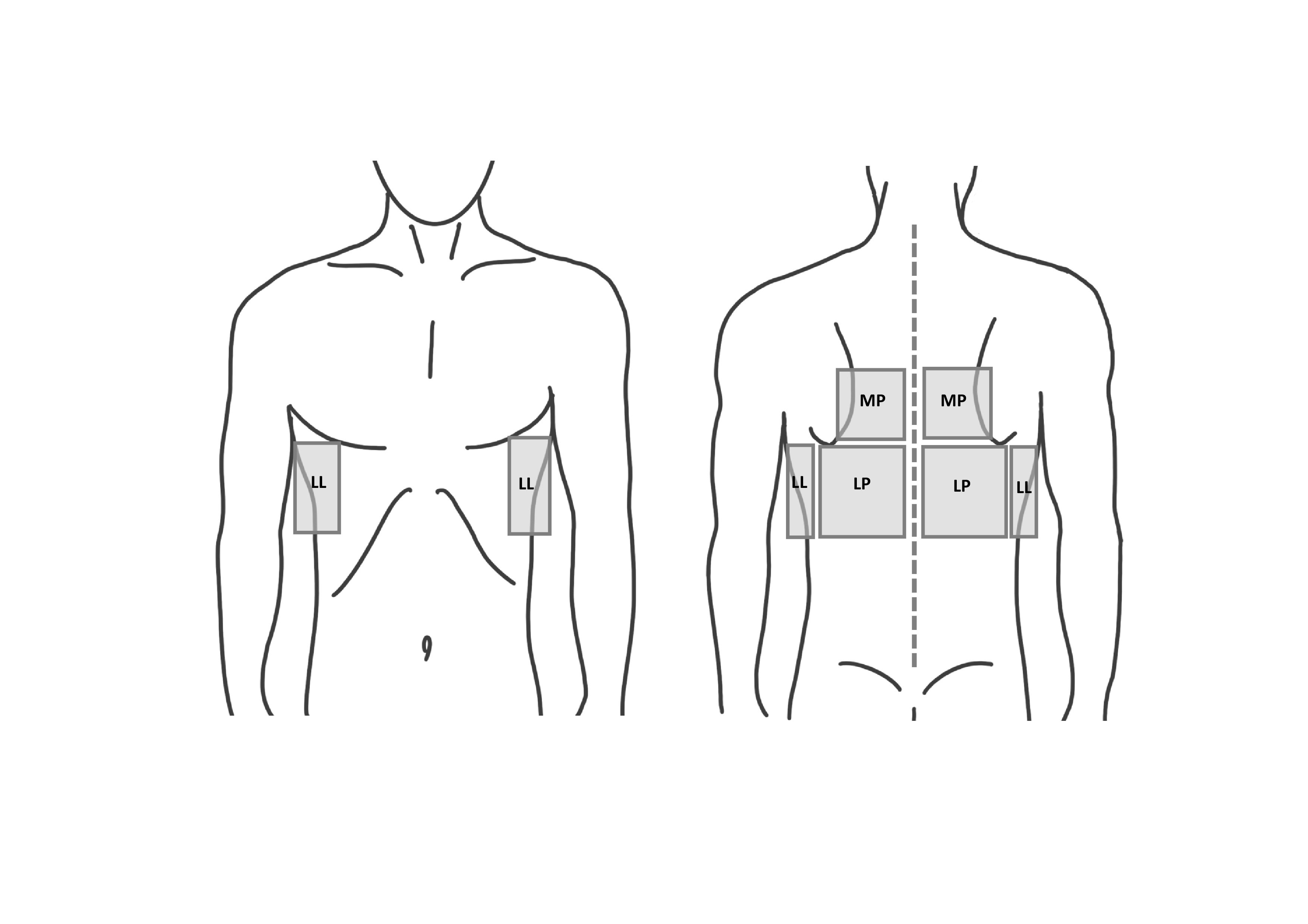

Supplement: Supplementary file 1 — Figure S1. Chest and back subdivisions of patients undergoing lung ultrasound in this study. LL, lower lateral (lower part of the lateral thorax between the anterior axillary line and the posterior axillary line); LP, lower posterior (lower part of the back below the inferior angle of the scapula and dorsal to the posterior axillary line); MP, middle posterior (middle part of the back medial to the scapula) [file GGI-21-1118-s006.tiff]

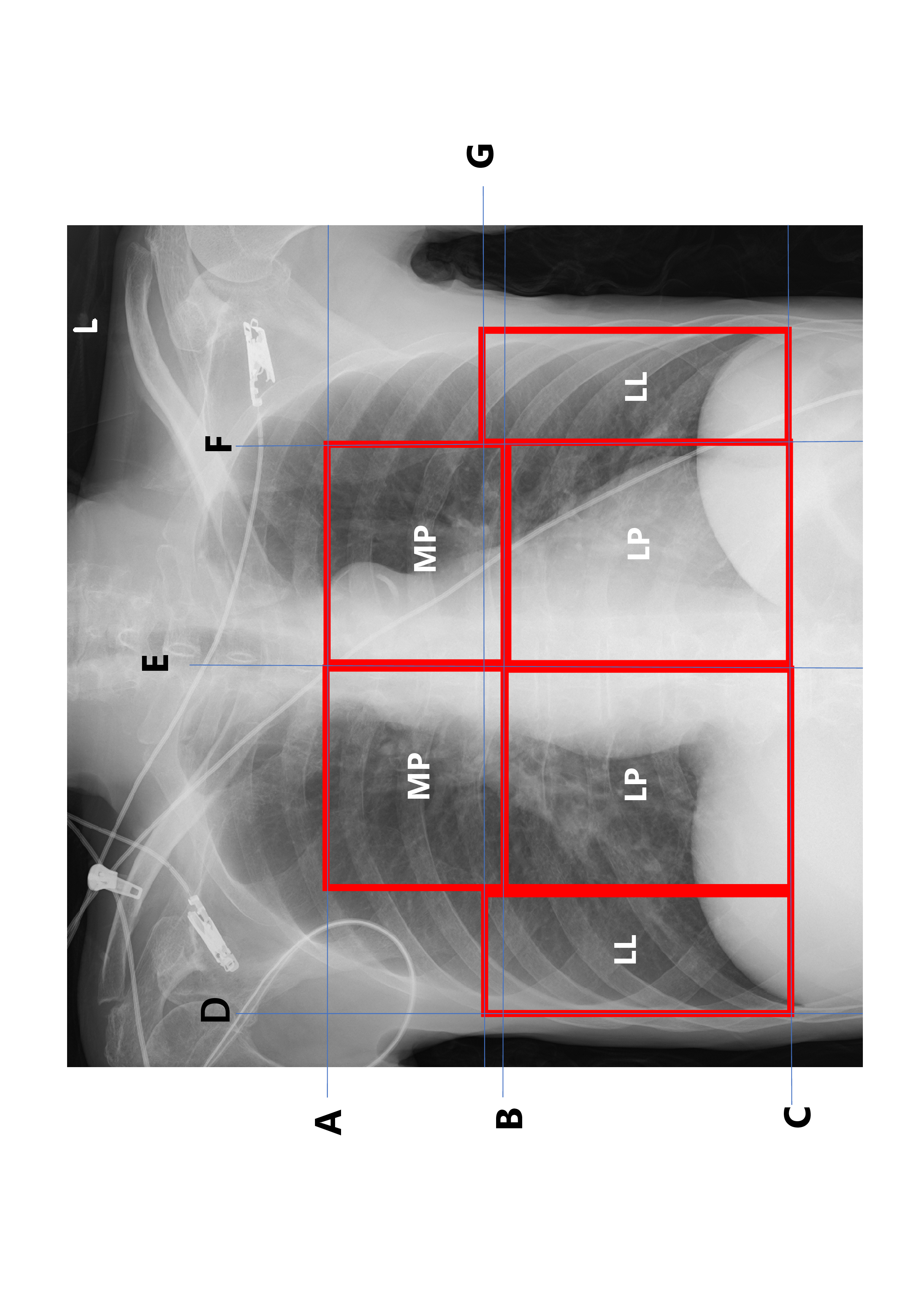

Supplement: Supplementary file 2 — Figure S2. Chest subdivisions of patients undergoing simple chest radiograph in this study. A, Line running across the middle of the scapula. B, Line running through the lower edge of the scapula. C, Line running through the costophrenic angle. D, Line running along the most lateral rib. E, Midline of the thoracic vertebrae F, Line running through the lateral third of the lung. G, Line running through half the height of the lung. [file GGI-21-1118-s003.tiff]

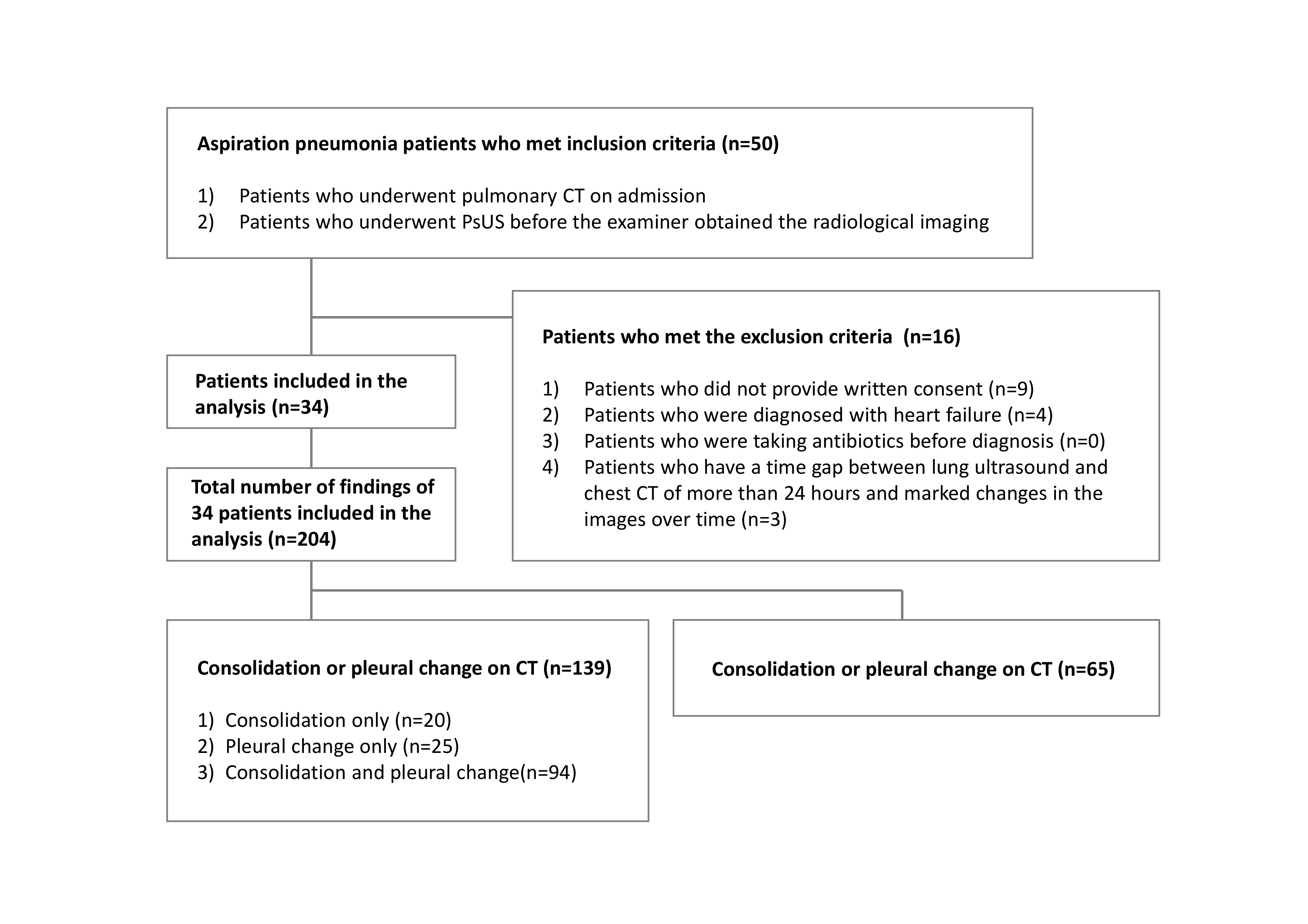

Supplement: Supplementary file 3 — Figure S3. Flow diagram of the study population [file GGI-21-1118-s004.tiff]
