# Supplementary material for: Diagnostic accuracy of pocket‐sized ultrasound for aspiration pneumonia in elderly patients without heart failure: A prospective observational study
Source: Geriatr Gerontol Int. 2021 Oct 14;21(12):1118–24. doi: 10.1111/ggi.14293 (PMC9293111; doi:10.1111/ggi.14293)
Supplement: Supplementary file 4 — Table S1. (a) Diagnostic accuracy of a combination of ultrasound findings (B‐line and US‐consolidation and pleural effusion) for suggesting CT‐consolidation. (b) Diagnostic accuracy of a combination of ultrasound findings (B‐line and US‐consolidation and pleural effusion) for suggesting pleural change on chest CT. (c) Diagnostic accuracy of a combination of ultrasound findings (B‐line and US‐consolidation and pleural effusion) for suggesting CT‐consolidation or pleural change on chest CT. [file GGI-21-1118-s001.docx]

**Supporting information**

**Table S1a** Diagnostic accuracy of a combination of ultrasound findings (B-line and US-consolidation and pleural effusion) for suggesting CT-consolidation

|  | **Sn**  **(95% CI)** | **Sp**  **(95% CI)** | **LR+**  **(95% CI)** | **LR−**  **(95% CI)** |
| --- | --- | --- | --- | --- |
| **Combination of ultrasound findings (and)** |  |  |  |  |
| B-line≥1 and US-consolidation | 0.368  (0.280-0.464) | 0.933  (0.861-0.975) | 5.526  (2.460-12.417) | 0.677  (0.582-0.787) |
| B-line≥3 and US-consolidation | 0.307  (0.224-0.400) | 0.956  (0.890-0.988) | 6.908  (2.549-18.719) | 0.725  (0.637-0.826) |
| B-line≥5 and US-consolidation | 0.211  (0.140-0.297) | 1.000  (0.940-1.000) | Inf  (NaN-Inf) | 0.789  (0.718-0.868) |
| B-line≥1 and effusion | 0.272  (0.193-0.363) | 0.944  (0.875-0.982) | 4.895  (1.984-12.078) | 0.771  (0.682-0.872) |
| B-line≥3 and effusion | 0.228  (0.155-0.316) | 0.967  (0.906-0.993) | 6.842  (2.139-21.885) | 0.799  (0.718-0.889) |
| B-line≥5 and effusion | 0.149  (0.089-0.228) | 0.978  (0.922-0.997) | 6.711  (1.592-28.291) | 0.870  (0.801-0.945) |
| B-line≥1 and US-consolidation and effusion | 0.202  (0.132-0.287) | 0.989  (0.940-1.000) | 18.158  (2.500-131.902) | 0.807  (0.734-0.888) |
| B-line≥3 and US-consolidation and effusion | 0.184  (0.118-0.268) | 0.989  (0.940-1.000) | 16.579  (2.273-120.916) | 0.825  (0.754-0.903) |
| B-line≥5 and US-consolidation and effusion | 0.140  (0.082-0.218) | 1.000  (0.940-1.000) | Inf  (NaN-Inf) | 0.860  (0.798-0.926) |
| **Combination of ultrasound findings (or)** |  |  |  |  |
| B-line≥1 or US-consolidation | 0.904  (0.834-0.951) | 0.422  (0.319-0.531) | 1.564  (1.298-1.884) | 0.229  (0.124-0.421) |
| B-line≥3 or US-consolidation | 0.781  (0.694-0.853) | 0.756  (0.654-0.840) | 3.194  (2.193-4.652) | 0.290  (0.201-0.418) |
| B-line≥5 or US-consolidation | 0.667  (0.572-0.752) | 0.811  (0.715-0.886) | 3.529  (2.256-5.521) | 0.411  (0.311-0.543) |
| B-line≥1 or effusion | 0.825  (0.742-0.889) | 0.467  (0.361-0.575) | 1.546  (1.252-1.909) | 0.376  (0.238-0.593) |
| B-line≥3 or effusion | 0.684  (0.591-0.768) | 0.800  (0.702-0.877) | 3.421  (2.222-5.267) | 0.395  (0.296-0.527) |
| B-line≥5 or effusion | 0.553  (0.457-0.646) | 0.889  (0.805-0.945) | 4.974  (2.710-9.129) | 0.503  (0.405-0.625) |
| B-line≥1 or US-consolidation or effusion | 0.904  (0.834-0.951) | 0.422  (0.319-0.531) | 1.564  (1.298-1.884) | 0.229  (0.124-0.421) |
| B-line≥3 or US-consolidation or effusion | 0.807  (0.723-0.875) | 0.733  (0.630-0.821) | 3.026  (2.124-4.312) | 0.263  (0.177-0.391) |
| B-line≥5 or US-consolidation or effusion | 0.728  (0.637-0.807) | 0.789  (0.690-0.868) | 3.449  (2.278-5.222) | 0.345  (0.251-0.474) |
| CI, confidence interval; CT, computed tomography; Inf, infinity; NaN, not a number; Sn, sensitivity; Sp, specificity | | | | |

**Table S1b** Diagnostic accuracy of a combination of ultrasound findings (B-line and US-consolidation and pleural effusion) for suggesting pleural change on chest CT

|  | **Sn**  **(95% CI)** | **Sp**  **(95% CI)** | **LR+**  **(95% CI)** | **LR−**  **(95% CI)** |
| --- | --- | --- | --- | --- |
| **Combination of ultrasound findings (and)** |  |  |  |  |
| B-line≥1 and US-consolidation | 0.353  (0.268-0.446) | 0.929  (0.853-0.974) | 5.000  (2.227-11.227) | 0.696  (0.602-0.805) |
| B-line≥3 and US-consolidation | 0.294  (0.214-0.385) | 0.953  (0.884-0.987) | 6.250  (2.308-16.927) | 0.741  (0.654-0.840) |
| B-line≥5 and US-consolidation | 0.185  (0.120-0.266) | 0.976  (0.918-0.997) | 7.857  (1.898-32.523) | 0.835  (0.762-0.915) |
| B-line≥1 and effusion | 0.286  (0.207-0.376) | 0.976  (0.918-0.997) | 12.143  (2.998-49.176) | 0.731  (0.650-0.823) |
| B-line≥3 and effusion | 0.235  (0.162-0.322) | 0.988  (0.936-1.000) | 20.000  (2.775-144.153) | 0.774  (0.699-0.857) |
| B-line≥5 and effusion | 0.160  (0.099-0.238) | 1.000  (0.937-1.000) | Inf  (NaN-Inf) | 0.840  (0.777-0.909) |
| B-line≥1 and US-consolidation and effusion | 0.193  (0.127-0.276) | 0.988  (0.936-1.000) | 16.429  (2.262-119.307) | 0.816  (0.745-0.894) |
| B-line≥3 and US-consolidation and effusion | 0.176  (0.113-0.257) | 0.988  (0.936-1.000) | 15.000  (2.057-109.370) | 0.833  (0.764-0.908) |
| B-line≥5 and US-consolidation and effusion | 0.134  (0.079-0.209) | 1.000  (0.937-1.000) | Inf  (NaN-Inf) | 0.866  (0.806-0.929) |
| **Combination of ultrasound findings (or)** |  |  |  |  |
| B-line≥1 or US-consolidation | 0.908  (0.841-0.953) | 0.447  (0.339-0.559) | 1.641  (1.344-2.004) | 0.207  (0.112-0.381) |
| B-line≥3 or US-consolidation | 0.832  (0.752-0.894) | 0.859  (0.766-0.925) | 5.893  (3.467-10.017) | 0.196  (0.130-0.295) |
| B-line≥5 or US-consolidation | 0.681  (0.589- 0.763) | 0.859  (0.766-0.925) | 4.821  (2.814-8.262) | 0.372  (0.282-0.490) |
| B-line≥1 or effusion | 0.832  (0.752-0.894) | 0.494  (0.384-0.605) | 1.645  (1.313-2.060) | 0.340  (0.216-0.536) |
| B-line≥3 or effusion | 0.748  (0.660-0.823) | 0.918  (0.838-0.966) | 9.082  (4.433-18.607) | 0.275  (0.200-0.377) |
| B-line≥5 or effusion | 0.563  (0.469-0.654) | 0.929  (0.853-0.974) | 7.976  (3.629-17.530) | 0.470  (0.380-0.581) |
| B-line≥1 or US-consolidation or effusion | 0.908  (0.841-0.953) | 0.447  (0.339-0.559) | 1.641  (1.344-2.004) | 0.207  (0.112-0.381) |
| B-line≥3 or US-consolidation or effusion | 0.866  (0.791-0.921) | 0.847  (0.753-0.916) | 5.659  (3.414-9.380) | 0.159  (0.100-0.253) |
| B-line≥5 or US-consolidation or effusion | 0.748  (0.660-0.823) | 0.847  (0.753-0.916) | 4.890  (2.933-8.152) | 0.298  (0.216-0.411) |
| CI, confidence interval; CT, computed tomography; Inf, infinity; NaN, not a number; Sn, sensitivity; Sp, specificity | | | | |

**Table S1c** Diagnostic accuracy of a combination of ultrasound findings (B-line and US-consolidation and pleural effusion) for suggesting CT-consolidation or pleural change on chest CT

|  | **Sn**  **(95% CI)** | **Sp**  **(95% CI)** | **LR+**  **(95% CI)** | **LR−**  **(95% CI)** |
| --- | --- | --- | --- | --- |
| **Combination of ultrasound findings (and)** |  |  |  |  |
| B-line≥1 and US-consolidation | 0.338  (0.260-0.423) | 0.985  (0.917-1.000) | 21.978  (3.100-155.827) | 0.672  (0.595-0.760) |
| B-line≥3 and US-consolidation | 0.273  (0.201-0.355) | 0.985  (0.917-1.000) | 17.770  (2.494-126.611) | 0.738  (0.663-0.821) |
| B-line≥5 and US-consolidation | 0.173  (0.114-0.246) | 1.000  (0.918-1.000) | Inf  (NaN-Inf) | 0.827  (0.767-0.893) |
| B-line≥1 and effusion | 0.252  (0.182-0.332) | 0.985  (0.917-1.000) | 16.367  (2.292-116.873) | 0.760  (0.687-0.841) |
| B-line≥3 and effusion | 0.201  (0.138-0.278) | 0.985  (0.917-1.000) | 13.094  (1.821-94.152) | 0.811  (0.742-0.886) |
| B-line≥5 and effusion | 0.137  (0.084-0.205) | 1.000  (0.918-1.000) | Inf  (NaN-Inf) | 0.863  (0.808-0.922) |
| B-line≥1 and US-consolidation and effusion | 0.165  (0.108-0.238) | 0.985  (0.917-1.000) | 10.755  (1.484-77.925) | 0.848  (0.782-.918) |
| B-line≥3 and US-consolidation and effusion | 0.151  (0.096-0.222) | 0.985  (0.917-1.000) | 9.820  (1.350-71.435) | 0.862  (0.799-0.931) |
| B-line≥5 and US-consolidation and effusion | 0.115  (0.067-0.180) | 1.000  (0.918-1.000) | Inf  (NaN-Inf) | 0.885  (0.833-0.940) |
| **Combination of ultrasound findings (or)** |  |  |  |  |
| B-line≥1 or US-consolidation | 0.878  (0.811-0.927) | 0.492  (0.366-0.619) | 1.729  (1.350-2.214) | 0.248  (0.149-0.413) |
| B-line≥3 or US-consolidation | 0.755  (0.675-0.824) | 0.908  (0.810-0.965) | 8.183  (3.796-17.642) | 0.269  (0.199-0.365) |
| B-line≥5 or US-consolidation | 0.626  (0.540-0.706) | 0.908  (0.810-0.965) | 6.781  (3.130-14.690) | 0.412  (0.328-0.518) |
| B-line≥1 or effusion | 0.806  (0.730-0.868) | 0.538  (0.410-0.663) | 1.746  (1.326-2.298) | 0.361  (0.240-0.542) |
| B-line≥3 or effusion | 0.669  (0.584-0.746) | 0.954  (0.871-0.990) | 14.496  (4.771-44.046) | 0.347  (0.272-0.442) |
| B-line≥5 or effusion | 0.504  (0.418-0.589) | 0.954  (0.871-0.990) | 10.911  (3.569-33.355) | 0.520  (0.437-0.620) |
| B-line≥1 or US-consolidation or effusion | 0.878  (0.811-0.927) | 0.492  (0.366-0.619) | 1.729  (1.350-2.214) | 0.248  (0.149-0.413) |
| B-line≥3 or US-consolidation or effusion | 0.791  (0.714-0.856) | 0.908  (0.810-0.965) | 8.573  (3.981-18.462) | 0.230  (0.165-0.321) |
| B-line≥5 or US-consolidation or effusion | 0.691  (0.607-0.766) | 0.908  (0.810-0.965) | 7.482  (3.463-16.166) | 0.341  (0.263-0.442) |
| CI, confidence interval; CT, computed tomography; Inf, infinity; NaN, not a number; Sn, sensitivity; Sp, specificity; LR+, positive likelihood ratio; LR−, negative likelihood ratio | | | | |
