# Supplementary material for: Diagnostic accuracy of pocket‐sized ultrasound for aspiration pneumonia in elderly patients without heart failure: A prospective observational study
Source: Geriatr Gerontol Int. 2021 Oct 14;21(12):1118–24. doi: 10.1111/ggi.14293 (PMC9293111; doi:10.1111/ggi.14293)
Supplement: Supplementary file 5 — Table S2. Diagnostic accuracy of chest radiography for CT findings (CT‐consolidation and pleural change). [file GGI-21-1118-s002.docx]

**Supporting information**

**Table S2** Diagnostic accuracy of chest radiography for CT findings (CT-consolidation and pleural change)

| **CT finding** | **Sn**  **(95% CI)** | **Sp**  **(95% CI)** | **LR+**  **(95% CI)** | **LR−**  **(95% CI)** |
| --- | --- | --- | --- | --- |
| **CT-consolidation** | 0.761  (0.672-0.836) | 0.482  (0.373-0.593) | 1.470  (1.168-1.850) | 0.495  (0.333-0.736) |
| **Pleural change** | 0.754  (0.666-0.829) | 0.488  (0.374-0.602) | 1.472  (1.161-1.866) | 0.504  (0.342-0.743) |
| **CT-consolidation or pleural change** | 0.739  (0.658-0.810) | 0.533  (0.400-0.663) | 1.584  (1.187-2.113) | 0.489  (0.339-0.706) |
| CI, confidence interval; CT, computed tomography; Sn, sensitivity; Sp, specificity; LR+, positive likelihood ratio; LR−, negative likelihood ratio | | | | |
