# Supplementary material for: Diagnostic accuracy of pocket‐sized ultrasound for aspiration pneumonia in elderly patients without heart failure: A prospective observational study
Source: Geriatr Gerontol Int. 2021 Oct 14;21(12):1118–24. doi: 10.1111/ggi.14293 (PMC9293111; doi:10.1111/ggi.14293)
Supplement: Supplementary file 6 — Table S3. (a) Diagnostic accuracy of a combination of simple chest radiography and ultrasound findings (B‐line and US‐consolidation and pleural effusion) for CT‐consolidation (b) Diagnostic accuracy of a combination of simple chest radiography and ultrasound findings (B‐line and US‐consolidation and pleural effusion) for pleural change on chest CT. (c) Diagnostic accuracy of a combination of simple chest radiography and ultrasound findings (B‐line and US‐consolidation and pleural effusion) for CT‐consolidation or pleural change on chest CT. [file GGI-21-1118-s005.docx]

**Supporting information**

**Table S3a** Diagnostic accuracy of a combination of simple chest radiography and ultrasound findings (B-line and US-consolidation and pleural effusion) for CT-consolidation

|  | **Sn**  **(95% CI)** | **Sp**  **(95% CI)** | **LR+**  **(95% CI)** | **LR−**  **(95% CI)** |
| --- | --- | --- | --- | --- |
| **Combination of radiograph and ultrasound findings (and)** |  |  |  |  |
| Abnormal radiograph and B-line≥1 | 0.558  (0.461-0.651) | 0.718  (0.610-0.810) | 1.975  (1.355-2.878) | 0.617  (0.482-0.789) |
| Abnormal radiograph and B-line≥3 | 0.434  (0.341-0.530) | 0.918  (0.838-0.966) | 5.265  (2.512-11.039) | 0.617  (0.519-0.734) |
| Abnormal radiograph and B-line≥5 | 0.283  (0.202-0.376) | 0.965  (0.900-0.993) | 8.024  (2.542-25.327) | 0.743  (0.657-0.840) |
| Abnormal radiograph and US-consolidation | 0.425  (0.332-0.521) | 0.941  (0.868-0.981) | 7.221  (3.004-17.358) | 0.611  (0.517-0.722) |
| Abnormal radiograph and B-line≥1 and US-consolidation | 0.292  (0.210-0.385) | 0.976  (0.918-0.997) | 12.412  (3.063-50.294) | 0.725  (0.641-0.820) |
| Abnormal radiograph and B-line≥3 and US-consolidation | 0.257  (0.179-0.347) | 1.000  (0.937-1.000) | Inf  (NaN-Inf) | 0.743  (0.667-0.828) |
| Abnormal radiograph and B-line≥5 and US-consolidation | 0.186  (0.119-0.270) | 1.000  (0.937-1.000) | Inf  (NaN-Inf) | 0.814  (0.746-0.889) |
| Abnormal radiograph and B-line≥1 and US-consolidation and effusion | 0.159  (0.097-0.240) | 1.000  (0.937-1.000) | Inf  (NaN-Inf) | 0.841  (0.776-0.911) |
| Abnormal radiograph and B-line≥3 and US-consolidation and effusion | 0.159  (0.097-0.240) | 1.000  (0.937-1.000) | Inf  (NaN-Inf) | 0.841  (0.776-0.911) |
| Abnormal radiograph and B-line≥5 and US-consolidation and effusion | 0.133  (0.076-0.209) | 1.000  (0.937-1.000) | Inf  (NaN-Inf) | 0.867  (0.807-0.932) |
| **Combination of radiograph and ultrasound findings (or)** |  |  |  |  |
| Abnormal radiograph or B-line≥1 | 0.929  (0.865-0.969) | 0.235  (0.150-0.340) | 1.215  (1.069-1.382) | 0.301  (0.139-0.650) |
| Abnormal radiograph or B-line≥3 | 0.867  (0.791-0.924) | 0.400  (0.295-0.512) | 1.445  (1.198-1.744) | 0.332  (0.194-0.569) |
| Abnormal radiograph or B-line≥5 | 0.814  (0.730-0.881) | 0.459  (0.350-0.570) | 1.504  (1.214-1.865) | 0.405  (0.258-0.635) |
| Abnormal radiograph or US-consolidation | 0.885  (0.811-0.937) | 0.400  (0.295-0.512) | 1.475  (1.225-1.776) | 0.288  (0.162-0.511) |
| Abnormal radiograph or B-line≥1 or US-consolidation | 0.973  (0.924-0.994) | 0.200  (0.121-0.301) | 1.217  (1.089-1.359) | 0.133  (0.040-0.438) |
| Abnormal radiograph or B-line≥3 or US-consolidation | 0.938  (0.877-0.975) | 0.365  (0.263-0.476) | 1.477  (1.248-1.747) | 0.170  (0.079-0.367) |
| Abnormal radiograph or B-line≥5 or US-consolidation | 0.912  (0.843-0.957) | 0.376  (0.274-0.488) | 1.462  (1.227-1.741) | 0.235  (0.122-0.451) |
| Abnormal radiograph or B-line≥1 or US-consolidation or effusion | 0.973  (0.924-0.994) | 0.200  (0.121-0.301) | 1.217  (1.089-1.359) | 0.133  (0.040-0.438 |
| Abnormal radiograph or B-line≥3 or US-consolidation or effusion | 0.947  (0.888-0.980) | 0.353  (0.252-0.464) | 1.463  (1.243-1.722) | 0.150  (0.066-0.345) |
| Abnormal radiograph or B-line≥5 or US-consolidation or effusion | 0.929  (0.865-0.969) | 0.365  (0.263-0.476) | 1.463  (1.235-1.732) | 0.194  (0.094-0.401) |
| CI, confidence interval; CT, computed tomography; Inf, infinity; NaN, not a number; Sn, sensitivity; Sp, specificity | | | | |

**Table S3b** Diagnostic accuracy of a combination of simple chest radiography and ultrasound findings (B-line and US-consolidation and pleural effusion) for pleural change on chest CT

|  | **Sn**  **(95% CI)** | **Sp**  **(95% CI)** | **LR+**  **(95% CI)** | **LR−**  **(95% CI)** |
| --- | --- | --- | --- | --- |
| **Combination of radiograph and ultrasound findings (and)** |  |  |  |  |
| Abnormal radiograph and B-line≥1 | 0.551  (0.457-0.643) | 0.725  (0.614-0.819) | 2.003  (1.354-2.962) | 0.620  (0.487-0.788) |
| Abnormal radiograph and B-line≥3 | 0.458  (0.366-0.552) | 0.975  (0.913-0.997) | 18.305  (4.594-72.943) | 0.556  (0.470-0.659) |
| Abnormal radiograph and B-line≥5 | 0.288  (0.208-0.379) | 0.988  (0.932-1.000) | 23.051  (3.220-164.995) | 0.721  (0.641-0.811) |
| Abnormal radiograph and US-consolidation | 0.407  (0.317-0.501) | 0.938  (0.860-0.979) | 6.508  (2.710-15.632) | 0.633  (0.539-0.742) |
| Abnormal radiograph and B-line≥1 and US-consolidation | 0.271  (0.193-0.361) | 0.962  (0.894-0.992) | 7.232  (2.292-22.814) | 0.757  (0.673-0.852) |
| Abnormal radiograph and B-line≥3 and US-consolidation | 0.229  (0.157-0.315) | 0.975  (0.913-0.997) | 9.153  (2.239-37.413) | 0.791  (0.713-0.878) |
| Abnormal radiograph and B-line≥5 and US-consolidation | 0.169  (0.107-0.250) | 0.988  (0.932-1.000) | 13.559  (1.857-99.016) | 0.841  (0.772-0.916) |
| Abnormal radiograph and B-line≥1 and US-consolidation and effusion | 0.153  (0.093-0.230) | 1.000  (0.933-1.000) | Inf  (NaN-Inf) | 0.847  (0.785-0.915) |
| Abnormal radiograph and B-line≥3 and US-consolidation and effusion | 0.153  (0.093-0.230) | 1.000  (0.933-1.000) | Inf  (NaN-Inf) | 0.847  (0.785-0.915) |
| Abnormal radiograph and B-line≥5 and US-consolidation and effusion | 0.127  (0.073-0.201) | 1.000  (0.933-1.000) | Inf  (NaN-Inf) | 0.873  (0.815-0.935) |
| **Combination of radiograph and ultrasound findings (or)** |  |  |  |  |
| Abnormal radiograph or B-line≥1 | 0.932  (0.871-0.970) | 0.250  (0.160-0.359) | 1.243  (1.085-1.423) | 0.271  (0.126-0.585) |
| Abnormal radiograph or B-line≥3 | 0.890  (0.819-0.940) | 0.450  (0.338-0.565) | 1.618  (1.314-1.992) | 0.245  (0.139-0.432) |
| Abnormal radiograph or B-line≥5 | 0.805  (0.722-0.872) | 0.462  (0.350-0.578) | 1.498  (1.200-1.870) | 0.421  (0.272-0.652) |
| Abnormal radiograph or US-consolidation | 0.881  (0.809-0.934) | 0.412  (0.304-0.528) | 1.500  (1.234-1.824) | 0.288  (0.165-0.502) |
| Abnormal radiograph or B-line≥1 or US-consolidation | 0.975  (0.927-0.995) | 0.212  (0.129-0.318) | 1.238  (1.100-1.392) | 0.120  (0.036-0.395) |
| Abnormal radiograph or B-line≥3 or US-consolidation | 0.949  (0.893-0.981) | 0.400  (0.292-0.516) | 1.582  (1.316-1.901) | 0.127  (0.056-0.290) |
| Abnormal radiograph or B-line≥5 or US-consolidation | 0.915  (0.850-0.959) | 0.400  (0.292-0.516) | 1.525  (1.265-1.839) | 0.212  (0.111-0.406) |
| Abnormal radiograph or B-line≥1 or US-consolidation or effusion | 0.975  (0.927-0.995) | 0.212  (0.129-0.318) | 1.238  (1.100-1.392) | 0.120  0.036-0.395) |
| Abnormal radiograph or B-line≥3 or US-consolidation or effusion | 0.966  (0.915-0.991) | 0.400  (0.292-0.516) | 1.610  (1.342-1.932) | 0.085  (0.031-0.230) |
| Abnormal radiograph or B-line≥5 or US-consolidation or effusion | 0.941  (0.882-0.976) | 0.400  (0.292-0.516) | 1.568  (1.304-1.886) | 0.148  (0.069-0.319) |
| CI, confidence interval; CT, computed tomography; Inf, infinity; NaN, not a number; Sn, sensitivity; Sp, specificity | | | | |

**Table S3c** Diagnostic accuracy of a combination of simple chest radiography and ultrasound findings (B-line and US-consolidation and pleural effusion) for CT-consolidation or pleural change on chest CT

|  | **Sn**  **(95% CI)** | **Sp**  **(95% CI)** | **LR+**  **(95% CI)** | **LR−**  **(95% CI)** |
| --- | --- | --- | --- | --- |
| **Combination of radiograph and ultrasound findings (and)** |  |  |  |  |
| Abnormal radiograph and B-line≥1 | 0.536  (0.449-0.621) | 0.783  (0.658-0.879) | 2.475  (1.493-4.103) | 0.592  (0.474-0.740) |
| Abnormal radiograph and B-line≥3 | 0.406  (0.323-0.493) | 1.000  (0.912-1.000) | Inf  (NaN-Inf) | 0.594  (0.518-0.682) |
| Abnormal radiograph and B-line≥5 | 0.254  (0.183-0.335) | 1.000  (0.912-1.000) | Inf  (NaN-Inf) | 0.746  (0.677-0.823) |
| Abnormal radiograph and US-consolidation | 0.370  (0.289-0.456) | 0.967  (0.885-0.996) | 11.087  (2.789-44.066) | 0.652  (0.569-0.747) |
| Abnormal radiograph and B-line≥1 and US-consolidation | 0.254  (0.183-0.335) | 1.000  (0.912-1.000) | Inf  (NaN-Inf) | 0.746  (0.677-0.823) |
| Abnormal radiograph and B-line≥3 and US-consolidation | 0.210  (0.145-0.288) | 1.000  (0.912-1.000) | Inf  (NaN-Inf) | 0.790  (0.725-0.861) |
| Abnormal radiograph and B-line≥5 and US-consolidation | 0.152  (0.097-0.223) | 1.000  (0.912-1.000) | Inf  (NaN-Inf) | 0.848  (0.790-0.910) |
| Abnormal radiograph and B-line≥1 and US-consolidation and effusion | 0.130  (0.079-0.198) | 1.000  (0.912-1.000) | Inf  (NaN-Inf) | 0.870  (0.815-0.928) |
| Abnormal radiograph and B-line≥3 and US-consolidation and effusion | 0.130  (0.079-0.198) | 1.000  (0.912-1.000) | Inf  (NaN-Inf) | 0.870  (0.815-0.928) |
| Abnormal radiograph and B-line≥5 and US-consolidation and effusion | 0.109  (0.062-0.173) | 1.000  (0.912-1.000) | Inf  (NaN-Inf) | 0.891  (0.841-0.945) |
| **Combination of radiograph and ultrasound findings (or)** |  |  |  |  |
| Abnormal radiograph or B-line≥1 | 0.920  (0.862-0.960) | 0.283  (0.175-0.414) | 1.284  (1.087-1.517) | 0.281  (0.140-0.564) |
| Abnormal radiograph or B-line≥3 | 0.862  (0.793-0.915) | 0.500  (0.368-0.632) | 1.725  (1.328-2.240) | 0.275  (0.169-0.449) |
| Abnormal radiograph or B-line≥5 | 0.790  (0.712-0.855) | 0.517  (0.384-0.648) | 1.634  (1.241-2.152) | 0.407  (0.271-0.610) |
| Abnormal radiograph or US-consolidation | 0.870  (0.802-0.921) | 0.483  (0.352-0.616) | 1.683  (1.307-2.168) | 0.270  (0.163-0.447) |
| Abnormal radiograph or B-line≥1 or US-consolidation | 0.964  (0.917-0.988) | 0.250  (0.147-0.379) | 1.285  (1.106-1.492) | 0.145  (0.055-0.381) |
| Abnormal radiograph or B-line≥3 or US-consolidation | 0.928  (0.871-0.965) | 0.467  (0.337-0.600) | 1.739  (1.366-2.214) | 0.155  (0.081-0.299) |
| Abnormal radiograph or B-line≥5 or US-consolidation | 0.899  (0.836-0.943) | 0.467  (0.337-0.600) | 1.685  (1.321-2.149) | 0.217  (0.124-0.383) |
| Abnormal radiograph or B-line≥1 or US-consolidation or effusion | 0.964  (0.917-0.988) | 0.250  (0.147-0.379) | 1.285  (1.106-1.492) | 0.145  (0.055-0.381) |
| Abnormal radiograph or B-line≥3 or US-consolidation or effusion | 0.942  (0.889-0.975) | 0.467  (0.337-0.600) | 1.766  (1.389-2.246) | 0.124  (0.060-0.256) |
| Abnormal radiograph or B-line≥5 or US-consolidation or effusion | 0.920  (0.862-0.960) | 0.467  (0.337-0.600) | 1.726  (1.355-2.197) | 0.171  (0.091-0.320) |
| CI, confidence interval; CT, computed tomography; Inf, infinity; NaN, not a number; Sn, sensitivity; Sp, specificity; LR+, positive likelihood ratio; LR−, negative likelihood ratio | | | | |
